# Renal tubule-specific *Atgl* deletion links kidney lipid metabolism to glucagon-like peptide 1 and insulin secretion independent of renal inflammation or lipotoxicity

**DOI:** 10.1016/j.molmet.2024.101887

**Published:** 2024-01-26

**Authors:** Maria F. Fernandes, Juan J. Aristizabal-Henao, Phillip M. Marvyn, Iman M'Hiri, Meghan A. Wiens, Monica Hoang, Manuel Sebastian, Renato Nachbar, Philippe St-Pierre, Kalsha Diaguarachchige De Silva, Geoffrey A. Wood, Jamie W. Joseph, Christine A. Doucette, André Marette, Ken D. Stark, Robin E. Duncan

**Affiliations:** 1 Department of Kinesiology and Health Sciences, University of Waterloo, Ontario, Canada; 2 School of Pharmacy, University of Waterloo, Ontario, Canada; 3 Max Rady College of Medicine, University of Manitoba, Manitoba, Canada; 4 Québec Heart and Lung Institute, Department of Medicine, Laval University, Québec, Canada; 5 Ontario Veterinary College, University of Guelph, Ontario, Canada

**Keywords:** Adipose triglyceride lipase (ATGL), Renal tubule cells, Lysophosphatidic acid, Glucagon-like peptide 1, Glucose-stimulated insulin secretion, Diabetes

## Abstract

**Objective:**

Lipotoxic injury from renal lipid accumulation in obesity and type 2 diabetes (T2D) is implicated in associated kidney damage. However, models examining effects of renal ectopic lipid accumulation independent of obesity or T2D are lacking. We generated renal tubule-specific adipose triglyceride lipase knockout (RT-SAKO) mice to determine if this targeted triacylglycerol (TAG) over-storage affects glycemic control and kidney health.

**Methods:**

Male and female RT-SAKO mice and their control littermates were tested for changes in glycemic control at 10–12 and 16–18 weeks of age. Markers of kidney health and blood lipid and hormone concentrations were analyzed. Kidney and blood lysophosphatidic acid (LPA) levels were measured, and a role for LPA in mediating impaired glycemic control was evaluated using the LPA receptor 1/3 inhibitor Ki-16425.

**Results:**

All groups remained insulin sensitive, but 16- to 18-week-old male RT-SAKO mice became glucose intolerant, without developing kidney inflammation or fibrosis. Rather, these mice displayed lower circulating insulin and glucagon-like peptide 1 (GLP-1) levels. Impaired first-phase glucose-stimulated insulin secretion was detected and restored by Exendin-4. Kidney and blood LPA levels were elevated in older male but not female RT-SAKO mice, associated with increased kidney diacylglycerol kinase epsilon. Inhibition of LPA-mediated signaling restored serum GLP-1 levels, first-phase insulin secretion, and glucose tolerance.

**Conclusions:**

TAG over-storage alone is insufficient to cause renal tubule lipotoxicity. This work is the first to show that endogenously derived LPA modulates GLP-1 levels *in vivo*, demonstrating a new mechanism of kidney-gut-pancreas crosstalk to regulate insulin secretion and glucose homeostasis.

## Highlights

• Renal-tubule cell triacylglycerol accumulation does not alone cause lipotoxicity.
• Renal adipose triglyceride lipase (ATGL) loss increases diacylglycerol kinase-ε.
• Renal ATGL loss increases kidney and blood lysophosphatidic acid levels.
• Endogenous lysophosphatidic acid (LPA) impairs GLP-1 secretion.
• LPA can mediate kidney-gut-pancreas cross-talk to impair glucose homeostasis.

## Abbreviations

AUC: area-under-the-curve
ATGL: adipose triglyceride lipase
Cd36: CD36 molecule
CKD: chronic kidney disease
Col1A1: collagen type I alpha 1 chain
Col1A4: collagen type IV alpha 1
DAG: diacylglycerol
Dgat1: diacylglycerol acyltransferase 1
Dgat2: diacylglycerol acyltransferase 2
DGK: diacylglycerol kinase
DM: diabetes mellitus
Enpp2: Autotaxin
ESRD: end-stage renal disease
Fabp4: fatty acid binding protein 4
Fas: fatty acid synthase
Fn: fibronectin
Gck: glucokinase
G6pc: glucose-6-phosphatase
GIP: gastric inhibitory polypeptide
GLP-1: glucagon-like peptide 1
Glut2: glucose transporter 2
GSIS: glucose-stimulated insulin secretion
GTT: glucose tolerance test
HIEC: hyperinsulinemic-euglycemic clamp
Hk1: hexokinase 1
Hk2: hexokinase 2
Hk3: hexokinase 3
HSL: hormone-sensitive lipase
iAOC: incremental area-over-the-curve
iAUC: incremental area-under-the-curve
Il1b: interleukin 1b
Il6: interleukin 6
iPLA2ζ: calcium-independent phospholipase A2 zeta
ITT: insulin tolerance test
Ki67: Antigen Ki67
LPA: lysophosphatidic acid
LPAR: lysophosphatidic acid receptor
Lpin1: lipin 1
MAG: monoacylglycerol
NEFA: non-esterified fatty acid
PNPLA2: patatin-like phospholipase A2
Pcna: proliferating cell nuclear antigen
Pdx1: pancreatic and duodenal homeobox 1
RT-SAKO: renal tubule-specific adipose triglyceride lipase knockout
Sglt1: sodium/glucose cotransporter protein 1
Sglt2: sodium/glucose cotransporter protein 2
TAG: triacylglycerol
T2D: type 2 diabetes
T2DM: type 2 diabetes mellitus
Tnf-α: tumor necrosis factor alpha

## 1 Introduction

Hyperglycemia has long been recognized as a risk factor for chronic kidney disease (CKD), but it is now emerging that subclinical [1,2] and overt [[3], [4], [5], [6]] kidney dysfunction also increase the risk for new onset diabetes mellitus (DM), indicating a vicious cycle. This relationship is poorly characterized [7], however, recent efforts have found direct mechanistic links. For example, blood urea elevation in mice has been shown to cause beta-islet cell oxidative stress and impaired insulin secretion [8]. Uremia, however, is characteristic of end-stage renal disease (ESRD), while CKD prior to ESRD is an independent risk factor for new-onset DM [5], indicating that additional and earlier factors are involved. Identifying the earliest underlying bioactive mediators that arise before either kidney damage or dysglycemia could provide new therapeutic targets to break this cycle at an early stage, as well as novel biomarkers to screen for those at greatest risk of developing both diseases. CKD and DM are major causes of morbidity and mortality in millions worldwide [9], and are also risk factors for a host of additional metabolic diseases [[10], [11], [12]], and therefore the potential benefit of understanding this relationship is significant.

To identify possible new mediators of the related risks of CKD and DM, we considered changes common to both diseases. Renal triacylglycerol (TAG) accumulation is a hallmark of both kidney injury and type 2 DM (T2DM) [[13], [14], [15], [16], [17], [18]]. It is also associated with risk factors common to both diseases [[19], [20], [21], [22], [23], [24], [25]], including rising adiposity [14,26] and advancing age [15,27]. Together, this suggests that renal steatosis may play a causal role in the vicious cycle between kidney disease and T2DM. In other glucoregulatory tissues such as liver and skeletal muscle, the inter-relationships between organ-specific steatosis, organ health, and glucose homeostasis have been well studied using tissue-specific genetic models [[28], [29], [30], [31]]. However, most data on renal steatosis, kidney health, and glycemic control have been derived from rodent overfeeding models [17,[32], [33], [34], [35]], or genetic models of generalized lipid overaccumulation, such as the *Adipose Triglyceride Lipase* (*Atgl*) whole-body knockout mouse model, which develops ectopic lipid deposits in all organs [36,37]. To overcome these confounding issues and allow for investigation of the effects of isolated renal steatosis on kidney health and glycemic control, we generated renal tubule-specific *Atgl* knockout (RT-SAKO) mice.

Renal tubule cells were chosen for targeting due to their role in the reuptake of albumin from the urinary filtrate, which makes them particularly susceptible to TAG over-storage. In the obese and insulin resistant states, circulating albumin can be saturated with non-esterified fatty acids (NEFA), and these can be used to synthesize excess TAG upon uptake by epithelial renal tubule cells [38]. *Atgl* (also called *Desnutrin* [39], *iPLA2ζ* [40], or *Pnpla2* [39]) was targeted since subsequent hydrolysis of TAG is required for export of NEFA back to the blood, and ATGL is highly expressed in kidney [39,41]. Indeed, we have previously reported that this lipase is concentrated in renal tubules near the lumen, where incoming NEFA concentrations are highest [42]. Thus, we hypothesized that impaired TAG breakdown in renal tubule cells of RT-SAKO mice would result in TAG over-storage, which would cause progressive lipotoxicity in renal tubules, followed by renal damage and inflammation that would initiate systemic insulin resistance and glucose intolerance [[43], [44], [45], [46]].

Although both male and female mice were normoglycemic at 10–12 weeks of age, and female mice retained normal glucose control throughout the study, impaired systemic glucose disposal developed by 16–18 weeks of age in male RT-SAKO mice. This occurred, however, in the absence of insulin resistance. Moreover, renal lipotoxicity and inflammation were not evident, and therefore not mechanistically implicated in this glucose intolerance. Therefore, we rejected our original hypothesis and searched for alternate mechanisms. Here, we describe a novel mechanism of kidney-gut-pancreas crosstalk, wherein kidney-derived lysophosphatidic acid (LPA) decreases glucagon-like peptide 1 (GLP-1) levels to impair glucose-stimulated insulin secretion (GSIS), triggering glucose intolerance.

## 2 Materials and methods

### 2.1 Animals

*Atgl flox/flox* mice with *LoxP* sites flanking the entire first exon of the *Atgl*/*Pnpla2* gene were a generous gift from Dr. Hei Sook Sul, and have been described previously [47,48]. RT-SAKO mice were generated by crossing *Atgl flox/flox mice* with Ksp1.3/Cre transgenic mice (B6.Cg-Tg(Cdh16-cre)91Igr/J) (The Jackson Laboratory, JAX stock #012237) [49] as described in Supplementary Methods S1, and were compared with littermate controls. Ksp1.3/Cre transgenic mice were chosen since they express Cre in renal tubule cells, but not in glomeruli or renal interstitial cells [49]. In studies using only wildtype mice, 18 week-old C57Bl/6J mice were used. Mice were housed in a temperature and humidity-controlled environment, on a 12:12 h light/dark cycle, and standard rodent chow and water were provided *ad libitum*. Mice were tested at two ages, 10–12 weeks and 16–18 weeks. Body weights and food intake were measured at each age, and mice were euthanized for organ dissection and weighing. Animal procedures were approved by the University of Waterloo Animal Care Committee (AUPP #13-19, #13-20, #17-16, #17-17, #43428, and #43325), and Comté de Protection des Animaux de L'Université Laval (CPAUL 2016-079-1) and comply with guidelines of the Canadian Council on Animal Care.

### 2.2 Drug treatments

Mice treated with Ki-16425 (Cayman Chemical, Ann Arbour, Michigan) at 5 mg/kg or vehicle control (10 % DMSO) received a single *i.p.* injection 30 min before glucose tolerance testing or insulin collection, as previously described by Rancoule et al. [50]. Mice treated with the GLP-1 receptor agonist Exendin-4 (Cedarlane, Mississauga, Ontario, Canada) at 5 μg/kg or vehicle control (10 % DMSO) received a single *i.p.* injection immediately before *i.p.* administration of glucose (1 g/kg). This dose of GLP-1 is in a range that has previously been used in studies with mice [[51], [52], [53]], and was determined in our work to be sufficient to significantly restore serum insulin levels in RT-SAKO mice without non-specifically elevating blood insulin levels in control mice.

### 2.3 Glucose sensitivity and insulin tolerance testing

Glucose tolerance tests (GTT) and insulin sensitivity tests (ITT) were performed as previously described, with minor modifications [54]. Briefly, for GTT mice were injected *i.p.* with 1 g/kg d-glucose following an overnight fast, and blood glucose was sampled from the tail using a FreeStyle Lite glucometer (Abbott Laboratories, Chicago, Illinois, U.S), and net incremental area-under-the-curve was calculated [55]. For ITT, mice were injected *i.p.* with insulin (0.74 U/kg) following a 4 h fast, and blood glucose concentrations were measured in samples collected from the tail vein, and net incremental area-over-the-curve was analyzed [55].

### 2.4 Hyperinsulinemic-euglycemic clamp

The hyperinsulinemic-euglycemic clamp (HIEC) was performed in catheterized 16–18 week old male RT-SAKO and control littermates, as previously described [56]. Briefly, the right jugular vein, and the left common carotid artery were catheterized for blood infusions and sampling, respectively. The insulin clamp procedure consisted of a 120 min constant infusion of insulin (2.5 mU/min/kg) and [3-^3^H]-glucose (0.67 μCi/min), where the glycemic response was monitored *via* blood sampling of the left common carotid artery. Infusion of 20 % dextrose was used as needed to maintain euglycaemia. Glucose metabolic fluxes were assessed using radiolabeled [3-^3^H]-glucose (0.67 μCi/min), to allow for the determination of hepatic glucose production and peripheral glucose uptake.

### 2.5 *In vivo* GSIS

Male RT-SAKO and control littermates, age 16–18 weeks, were fasted overnight prior to *i.p.* injection with d-glucose (3 g/kg). Blood was collected from the tail vein immediately before the glucose injection (time 0 min), and at 2 min and, where indicated, 20 min after glucose stimulation. Serum insulin was measured using a rat/mouse insulin ELISA kit (Cat# EZRMI-13K, Millipore-Sigma, Oakville, Ontario, Canada).

### 2.6 Islet isolation and *ex vivo* GSIS

Mouse islets were isolated and cultured for 2 h as previously described [[57], [58], [59], [60], [61]]. Briefly, the pancreas was perfused *via* the bile duct with Liberase TL and then incubated for 20 min at 37 °C. The digested pancreas was then passed through a sieve (Bellco, Vineland, NJ, USA) and washed three times with ice-cold HANKS buffer, followed by resuspension in RPMI 1640 media (Hyclone, Marlborough, MA, USA), containing 0.1 M HEPES, 10 % FBS, and 20 mM glutamine. Islet *ex vivo* GSIS was measured as previously described [57,58,61]. Briefly, 20 size-matched islets from RT-SAKO and control littermates were pre-incubated in Krebs–Ringer buffer (KRB) containing 2.8 mM glucose for 1 h at 37 °C/5 % CO2, to equilibrate. Islets were then incubated for an additional 30 min at 37 °C in KRB containing low glucose (2.8 mM) or high glucose (16.7 mM), or 300 mM potassium chloride (KCl), as previously described [62]. Insulin concentration was measured using a mouse insulin ELISA kit (Cat#80-INSMS-E01; ALPCO, Salem, New Hampshire, USA) and normalized to total RNA content.

### 2.7 Analysis of lysophosphatidic acid (LPA) levels

#### 2.7.1 Sample preparation and lipid extraction

Whole blood samples were collected by cardiac puncture into EDTA-lined vacutainers and centrifuged to obtain plasma that was stored at −80 °C. Kidneys were flash-frozen in liquid nitrogen and stored at −80 °C until analysis. Whole kidneys were homogenized in 500 μL buffer (0.1 M EDTA and 0.1 M sodium phosphate). Lipids were extracted from kidney homogenates and plasma samples using a butanol-based protocol as described previously [63]. Briefly, 1.5 mL buffer (30 mM citric acid and 40 mM disodium phosphate), 2 mL water-saturated butanol (9:1 butanol/water) and 4 mL n-butanol delivering 20 pmol of the internal standard (heptadecanoyl LPA, Millipore-Sigma, Oakville, Ontario, Canada) were added. Samples were vortexed and centrifuged at 2000× *g* for 15 min. The top organic layer was extracted and evaporated under nitrogen gas. Samples were then reconstituted in 100 μL of 3:1 methanol/isopropanol and stored at 4 °C until analysis.

#### 2.7.2 Targeted ultra-high performance liquid chromatography/tandem mass spectrometry (UHPLC-MS/MS)

LPA species were measured by UHPLC-MS/MS using a Waters Acquity UPLC system coupled to a Waters Synapt G2Si quadrupole/time-of-flight mass spectrometer (Waters Corporation, USA). The mobile phase consisted of A: 60:40 methanol/water and B: methanol, both with 5 mM ammonium formate and 0.1 % formic acid. The multi-step UHPLC gradient program has been described previously [63]. The mass spectrometer was operated using an optimized time-of-flight multiple reaction monitoring (ToF-MRM) method with electrospray ionization source voltage −2.0 kV, and used a targeted inclusion list for 6 analytes and the internal standard. Peak areas were integrated using Waters MassLynx software and were normalized relative to the peak area and amount of internal standard used. Kidney LPA levels were normalized to total protein concentrations and plasma levels were normalized by volume of plasma used.

### 2.8 Analysis of alpha (α) - and beta (β)-cell masses

Isolated pancreas from RT-SAKO and control littermates were fixed in Z-fix (Anatech Ltd, MI, USA) for 6 h and embedded in paraffin. Pancreas sections were prepared at three levels, each separated by 100 μm. Slides were stained for glucagon (1:500, Abcam, USA) and insulin (1:1000, Abcam, USA). The visualization system used was EnVision™ G|2 Doublestain System, Rabbit/Mouse (DAB+/Permanent Red) (Agilent, Canada). Immunohistochemistry was analyzed using an Aperio ImageScope (Leica Biosystems, USA) according to the manufacturer's instructions, for determining α-cell and β-cell masses.

### 2.9 Islet morphology

Islet morphology was analyzed as previously described [62]. Whole mouse pancreas embedded in OCT was cut into 10 μm cryosections, mounted on glass slides, fixed in 10 % formalin, blocked for 1 h in 5 % donkey serum and incubated overnight at 4 °C with primary antibodies (1:300 dilution) to insulin (Cat#4590) or glucagon (Cat#2760) from Cell Signaling Technology Inc., Whitby, Ontario, Canada). Sections were then incubated with Alexa Fluor™ 488-conjugated anti-rabbit secondary antibodies (1:500 dilution) for 1 h (Cat #711-545-152, Jackson ImmunoResearch Laboratories, West Grove, Pennsylvania, USA) and mounted in Vectashield mounting medium containing DAPI (Vector Laboratories, Newark, California, USA) for imaging with a Zeiss Axio Observer Z1 microscope at 20× magnification. Total islet area was calculated with Zen2 Pro software, and ImageJ software was used to quantify the number of insulin- and glucagon-positive cells as well as total cell number. The α-cell and β-cell fractions were calculated as the respective ratio of the number of cells stained positive for glucagon or insulin to the total number of nuclei in the islet.

### 2.10 Gene expression analysis

Gene expression analysis in various tissues was performed as we have previously described [64,65]. RNA was extracted using TRIzol reagent (Thermo Fisher Scientific, USA). Total RNA (2 μg) was reverse transcribed to cDNA using a high-capacity reverse transcription kit (Applied Biosystems, Waltham, Massachusetts, USA) according to the manufacturer's instructions. qPCR was performed using PerfeCTa SYBR Green Fastmix (Quantabio, Beverly, Massachusetts, USA). The relative gene expression was calculated by the change-in-threshold (−ΔΔCT) method, and expression of the gene of interest was normalized to expression of a housekeeping gene. Primer sequences are listed in the Supplementary Methods.

### 2.11 Circulating lipids and hormones

Serum levels of insulin, glucagon, gastric inhibitory polypeptide (GIP), leptin, resistin, and GLP-1 were measured in plasma isolated from mice that were fasted overnight using a multiplex bead-based assay performed on a Bio-Plex 200 system (Bio-Rad, Mississauga, ON, Canada) (Bio-Rad Laboratories, Missisissauga, Ontario, Canada) according to the manufacturer's instructions. Serum TAG, NEFA, and total cholesterol were measured using colorimetric assay kits (Wako Diagnostics, Richmond, VA, USA). Serum creatinine was measured using a colorimetric assay kit (Cayman Chemicals, Ann Arbour, MI, USA).

### 2.12 Immunoblotting

Mice were euthanized by cervical dislocation and organs were rapidly dissected and flash-frozen in liquid nitrogen. Samples were homogenized on ice in RIPA buffer (50 mM Tris, pH 7.4; 150 mM NaCl; 0.1 % SDS; 0.5 % sodium deoxycholate; 1× Triton ×100) with added protease inhibitor cocktail (Millipore-Sigma, Mississauga, Ontario, Canada) using a Polytron homogenizer. Homogenates were centrifuged for 15 min at 10,000 × *g*. Protein samples (12–15 μg) were separated by electrophoresis and electro-transferred to a nitrocellulose membrane (Bio-Rad, Mississauga, Ontario, Canada). Membranes were blocked in Tris-buffered saline with and 0.1 % Tween-20 and 5 % skim milk powder or 5 % bovine serum albumin, then incubated with: anti-ATGL (Cat #2138; 1:1000; Cell Signaling Technology Inc, USA); anti-DGKε (Cat #ab218039; 1:1000; Abcam, USA); anti-HSL (Cat #4107; 1:1000; Cell Signaling Technology Inc, USA); anti-GAPDH (Cat#5174; 1:1000; Cell Signaling Technology Inc, USA), followed by anti-rabbit Immunoglobulin G horseradish peroxidase-conjugate (Cat #7074; 1:10000; Cell Signaling Technology Inc, USA). Immunocomplexes were visualized by chemiluminescence using Luminata (Millipore-Sigma, Mississauga, Ontario, Canada), and band densities were quantified using ImageJ software. Data were normalized to GAPDH or total protein loading following visualization by Stain-Free™ gel imaging (Bio-Rad, Mississauga, Ontario, Canada).

### 2.13 Statistics

Student's T-test (2-tailed, unpaired) was used for comparisons between two groups. Two-way ANOVA with Tukey's multiple comparisons *post-hoc* test was used for multiple group comparisons. The results are expressed as means ± S.E.M. Statistical significance was considered at P ≤ 0.05. Significance is reported as: ∗P < 0.05; ∗∗P < 0.01; ∗∗∗P < 0.001, ∗∗∗∗P < 0.0001. Statistical analysis was performed using GraphPad Prism (GraphPad Software, Boston, MA, USA).

## 3 Results

### 3.1 RT-SAKO mice develop glucose intolerance in an age- and sex-dependent manner but remain insulin sensitive

RT-SAKO mice were generated (Figs. S1a–c). Since gene ablation was targeted to renal tubule cells, rather than the entire kidney, these mice expectedly displayed a significant, but not complete reduction in renal *Atgl* mRNA expression (by ∼1/3rd) and ATGL total protein levels (by ∼1/2) (Figs. S1d–f). Renal tubule TAG content was visibly increased in histochemical sections stained with Oil-Red-O, and quantitation of kidney TAG by thin layer chromatography coupled with gas chromatography indicated that an ∼2-fold increase in fatty acyl chain content had occurred (Figure S1g–h and Suppl. Methods). Gene expression and total protein levels of hormone sensitive lipase (HSL) did not differ between control and RT-SAKO mice (Figure S1i and j). There were no significant differences in body weight, food intake, or tissue masses between age- and sex-matched RT-SAKO mice and their littermate controls at either 10–12 or 16–18 weeks of age, highlighting the tissue-specificity of the model (Fig. S2). Of note, the lack of difference in kidney weights was not unexpected, since renal TAG content typically comprises only a small proportion of kidney mass (*i.e.* 1-2 %) and, therefore, even a doubling in TAG content was not expected to significantly alter the mass of this organ, although it was still expected to affect metabolic function [26].

**Figure 1:**
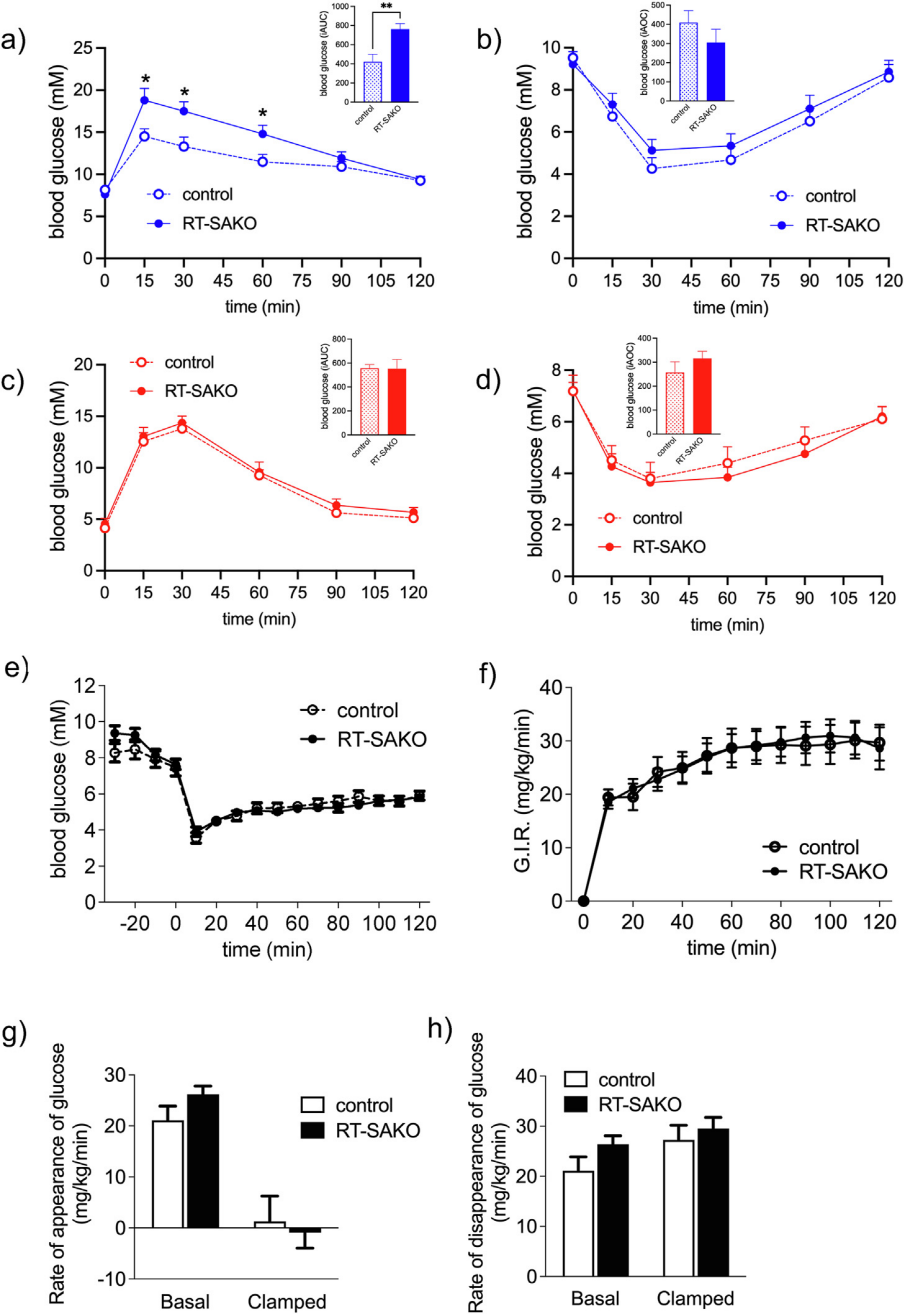
**Male RT-SAKO mice develop glucose intolerance by age 16**–**18 wks but remain insulin sensitive.** (A) Male RT-SAKO mice, age 16–18 weeks, develop glucose intolerance compared to their age-matched control littermates as demonstrated by glucose measures at individual timepoints, and as indicated in the overall analysis of the net incremental area under the curve (iAUC) for each mouse (inset) (n = 10–11). (B) Male RT-SAKO mice, age 16–18 weeks, do not exhibit insulin intolerance compared to age-matched littermate controls, either at specific timepoints following insulin administration or over the 2-hour response, as determined by analysis of net incremental area over the curve (iAOC) for each mouse (inset) (n = 14–15). (C) Female RT-SAKO mice, age 16–18 weeks, do not exhibit significant differences in glucose sensitivity compared to control littermates either at specific timepoints or based on iAUC analysis (n = 5). (D) Female RT-SAKO mice, age 16–18 weeks do not exhibit insulin resistance compared to control littermates either at specific timepoints tested or based on iAOC (n = 8–9). (E) Euglycemia was maintained in the hyperinsulinemic-euglycemic clamping study of male RT-SAKO and control littermate mice (16–18 weeks of age), and (F) the glucose infusion rate required did not differ significantly between these groups (n = 11–13). (G) The rate of appearance of glucose from the liver did not differ significantly between male RT-SAKO mice and their control littermates (16–18 weeks), either under basal conditions, or during the hyperinsulinemic-euglycemic clamp (n = 8–13). (h) The rate of disappearance of glucose from the liver did not differ significantly between genotypes under basal or clamped conditions (n = 8–13). Data are means ± SEM. ∗P < 0.05 versus littermate controls at the same timepoint, ∗∗P<0.01.

We initially hypothesized that TAG over-storage would cause a progressive lipotoxicity and inflammation resulting in insulin resistance and glucose intolerance. Analysis of glucose tolerance and insulin sensitivity in male and female RT-SAKO mice at 10–12 weeks of age indicated no significant differences from littermate controls (Fig. S3). Since we expected that a progressive pathology would be necessary to cause kidney damage and inflammation at a level sufficient to affect glycemic control, this was not unexpected. However, in contrast to our predictions, male RT-SAKO mice exhibited glucose intolerance at 16–18 weeks (Figure 1A), without decreased insulin sensitivity (Figure 1B). Although mean baseline glucose concentrations did not differ between genotypes, glucose concentrations at 15, 30, and 60 min were significantly greater in RT-SAKO mice than littermate controls, and the net incremental rise in blood glucose calculated as area-under-the-curve (iAUC) was close to double. Notably, this effect was sexually dimorphic, with female RT-SAKO mice demonstrating no difference in either glucose tolerance (Figure 1C) or insulin sensitivity (Figure 1D) relative to their littermate controls at this age.

Although systemic insulin responses did not differ significantly, we next performed HIEC on 16-to 18-week-old male RT-SAKO mice and their wildtype littermates to more closely examine whole-body, hepatic and peripheral insulin sensitivity (Figure 1E–H). In support of the general findings from the insulin sensitivity test (Figure 1B), blood glucose responses to infused insulin (Figure 1E), and the glucose infusion rate needed to maintain euglycemia throughout the insulin infusion (Figure 1F) were similar between RT-SAKO mice and control littermates. The rates of glucose appearance (mainly from the liver) (Figure 1G), and disappearance of glucose (mainly from skeletal muscle) (Figure 1H) in either basal or clamped conditions also did not differ significantly between genotypes. This indicated that alternate mechanisms, besides systemic insulin resistance or changes in hepatic or peripheral glucose handling, should be explored to explain the observed glucose intolerance in male mice.

### 3.2 Male RT-SAKO mice have lower serum insulin and GLP-1 levels and impaired GSIS, without evidence of renal lipotoxicity

**Figure 2:**
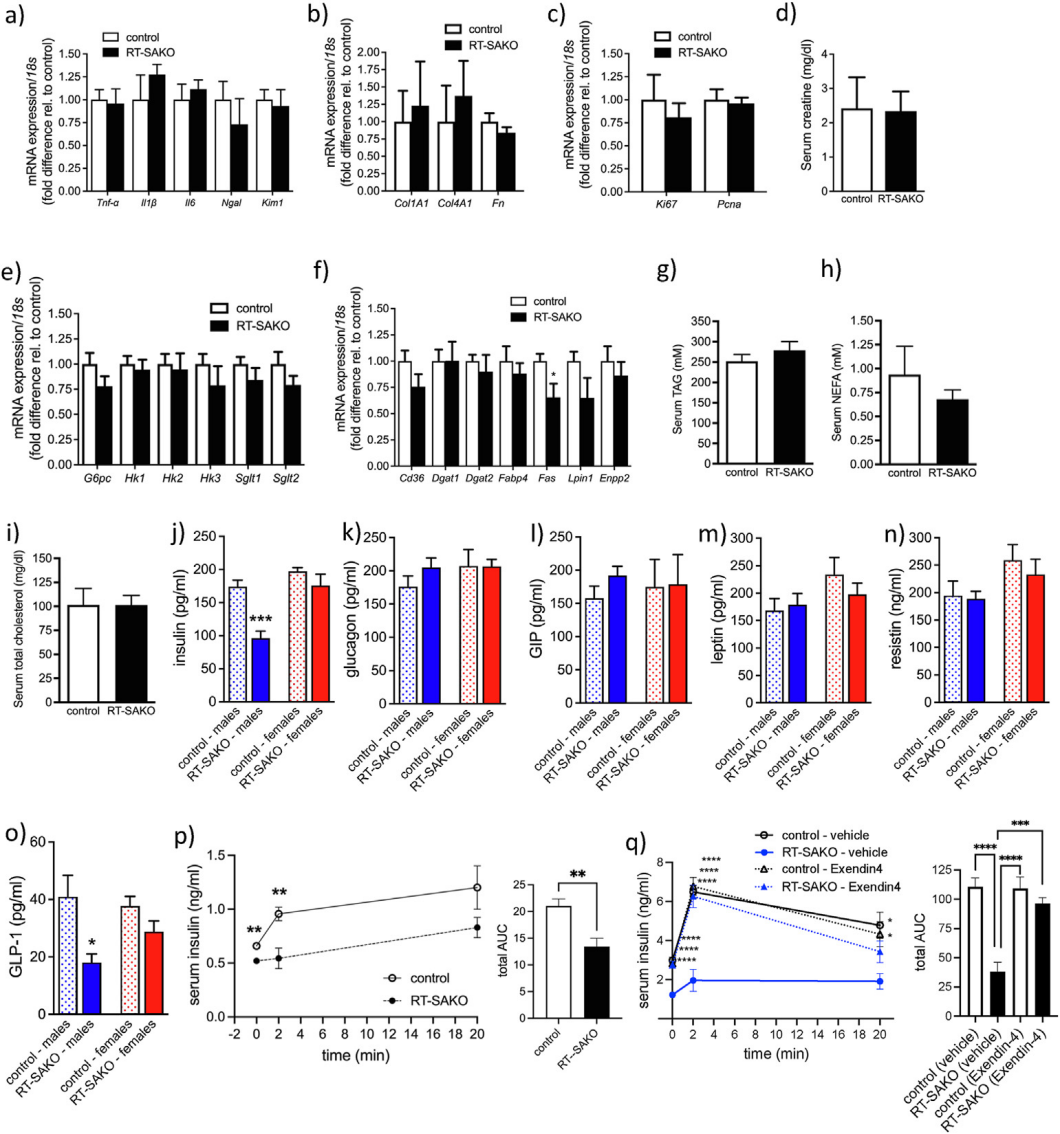
**Male RT-SAKO mice have lower serum insulin and GLP-1 levels and impaired GSIS, without renal lipotoxicity.** Renal gene expression was determined relative to *18s* as a loading control, and then expressed as fold-difference relative to control levels for genes involved in (A) inflammation (*i.e., tumor necrosis factor α* (*Tnf-α*), *interleukin 1b* (*Il1b*), and *interleukin 6* (*Il6*)) (n = 6–9) and kidney injury (*i.e., Neutrophil gelatinase-associated lipocalin (Ngal), Kidney injury molecule 1 (Kim1)*) (n = 5), (B) fibrosis (*i.e., collagen type I alpha 1 chain* (*Col1A1*), *collagen type IV alpha 1* (*Col1A4*), and *fibronectin* (*Fn*)) (n = 6–12), (C) proliferation and repair (*i.e., Antigen Ki67* (*Ki67*) and *proliferating cell nuclear antigen* (*Pcna*) (n = 11–12). Serum creatinine concentrations in male RT-SAKO mice and control littermates, ages 16–18 wks (n = 3–16) (D). Renal expression of genes involved in glucose metabolism (*i.e., glucose-6-phosphatase* (*G6pc*), *hexokinase 1* (*Hk1*), *hexokinase 2* (*Hk2*), *hexokinase 3* (*Hk3*), *sodium/glucose cotransporter protein 1* (*Sglt1*), and *sodium/glucose cotransporter protein 2* (*Sglt2*)) (n = 8–9) (E) and lipid metabolism (*i.e., CD36 molecule* (*Cd36*), *diacylglycerol acyltransferase 1* (*Dgat 1*), *diacylglycerol acyltransferase 2* (*Dgat 2*), *fatty acid binding protein 4* (*Fabp4*), *fatty acid synthase* (*Fas*), *lipin 1* (*Lpin1*), and *autotaxin* (*Enpp2*)) (n = 7–9) (F), in male RT-SAKO and control littermate mice, ages 16–18 wks. Serum concentrations of total triacylglycerol (TAG) (G), non-esterified fatty acids (NEFA) (H), and cholesterol (I) are shown (n = 3–5). Serum concentrations of insulin (J), glucagon (K), GIP (L), leptin (M), resistin (N) and GLP-1 (O) are shown for 16–18 wk old RT-SAKO and control littermate male (n = 7) and female (n = 5) mice. Data are means ± SEM. ∗P < 0.05, ∗∗∗P < 0.001 versus littermate controls of the same sex by Student's t-test. (P) Glucose-stimulated insulin secretion (GSIS) was analyzed by determining plasma insulin concentrations in male 16–18 wk old RT-SAKO mice and their control littermates immediately before glucose injection, and at 2 min and 20 min afterwards (left panel). Data are means ± SEM, ∗∗P < 0.01 versus control measures at the same timepoint by Student's t-test. Total AUC was calculated for the plasma insulin response (right panel). Data are means ± SEM, ∗∗P < 0.01 (n = 5). (Q) GSIS was determined in male control and RT-SAKO littermates by measuring plasma insulin concentrations after vehicle or Exendin4 administration but immediately before glucose injection (time 0) and at 2 min and 20 min after glucose injection (left panel). Data are means ± SEM, ∗P < 0.05, ∗∗∗∗P < 0.0001 versus RT-SAKO (vehicle) at the same timepoint, as determined by 2-way ANOVA with Tukey's post-hoc test (n = 5). Total AUC was calculated for the GSIS response (left panel), data are means ± SEM, ∗∗∗P < 0.001, ∗∗∗∗P < 0.0001 determined by 2-way ANOVA with Tukey's post-hoc test (n = 5).

We initially hypothesized that kidney lipotoxicity would result following the renal tubule-specific ablation of *Atgl*, and that related inflammation and fibrosis would be causal factors in dysregulated glucose control. We measured the gene expression of proinflammatory cytokines (*i.e. Tnf-α*, *Il1b*, and *Il6*) and markers of kidney injury (*i.e. Ngal, Kim-1*) in kidneys of male 16–18 wk old RT-SAKO mice and their control littermates and found that there was no significant difference in expression between the genotypes (Figure 2A). Additionally, there were no differences in the renal expression of genes involved in fibrosis (*i.e. Col1A1, Col4A1, Fn*) (Figure 2B) or genes involved in cell proliferation that could indicate differences in rates of cell turnover or repair in the kidneys (*i.e. Ki67*, *Pcna*) (Figure 2C). There was also no indication of altered renal function, with RT-SAKO and control littermates having similar concentrations of serum creatinine (Figure 2D), and microscopic evaluation of kidney slide sections (Suppl. Fig. 1h) by a veterinary pathologist indicated no evidence of histologically apparent lesions. Thus, in agreement with our finding of an absence of insulin resistance in this model, but in contrast to our original hypothesis, renal lipotoxicity, inflammation, and fibrosis did not appear to be mechanistically involved in the dysglycemia observed.

In addition to the liver, the kidneys are important regulators of blood glucose. Gluconeogenesis in the kidneys generates a quarter or more of blood glucose [66], while inhibition of the re-uptake of glucose from the urinary filtrate is a pharmacological target for the treatment of hyperglycemia in diabetes [67]. We examined the renal expression of a series of glucoregulatory genes for evidence of alterations. However, there were no significant differences in the expression of glucose-6-phosphatase needed for glucose release from cells to the bloodstream (*G6pc*), or any of the hexokinases involved in glucose trapping in kidney (*i.e., Hk1, Hk2, Hk3*), or in either of the sodium-dependent glucose transporters that modulate the re-uptake of glucose from the urinary filtrate (*i.e. Sglt1, Sglt2*) (Figure 2E). Examination of the expression of genes directly involved in fatty acid and TAG metabolism revealed only small differences between RT-SAKO and control littermates that suggested a possible minor compensatory down-regulation of *de novo* lipogenesis (Figure 2F), but did not suggest a mechanism to link altered renal TAG metabolism to glucose intolerance. Measures of major serum lipids, including TAG, NEFA, and total cholesterol, also indicated no significant differences between RT-SAKO and control littermates (Figure 2G–I), and therefore did not provide a mechanism to link renal steatosis in this model to the impaired glycemic control observed.

In the absence of evidence of a difference in tissue masses (Suppl. Fig. 2), hepatic (Figure 1E–H) or renal (Figure 2E) glucose handling, systemic or hepatic insulin resistance (Figure 1B, E–H) or renal inflammation or fibrosis (Figure 2A–C), we considered alternate hypotheses to explain the impaired glucose tolerance observed in 16- to 18-week-old male RT-SAKO mice. We examined serum concentrations of hormones involved in glycemic and metabolic regulation, which indicated two specific effects. First, serum insulin concentrations in fasted 16- to 18-wk-old male RT-SAKO mice were ∼45 % lower than concentrations in control littermates, while insulin concentrations in 16- to 18-wk old female RT-SAKO and control mice did not differ (Figure 2J). Second, while serum concentrations of glucagon, GIP, leptin, and resistin did not differ between sex-matched control and RT-SAKO mice (Figure 2K–N), serum levels of GLP-1 were significantly lower (by ∼56 %) in 16- to 18-wk-old male, but not female, RT-SAKO mice (Figure 2O). To test first- and second phase insulin secretory responses *in vivo*, serum insulin levels were measured immediately before a glucose injection, and at 2 min and 20 min following injection. Baseline blood insulin concentrations were found to be lower, and first phase *in vivo* GSIS was diminished in male RT-SAKO mice (Figure 2P, left panel). These mice also had a deficiency in the total insulinemic response recorded over 20 min, as determined by AUC evaluation (Figure 2P, right panel). GLP-1 is a potent incretin that is important in GSIS, particularly in the early or first-phase of insulin release [68]. The finding of lower GLP-1 concentrations in these mice therefore strongly suggested a possible role for this hormone in the reduced insulinemia evident in RT-SAKO mice. To test this, mice were pre-injected with the GLP-1 receptor agonist Exendin-4 immediately before glucose administration, to raise total GLP-1 activity. This was found to restore basal serum insulin concentrations and insulin concentrations at 2 min and 20 min post-glucose-injection in 16- to 18-wk-old male RT-SAKO mice to levels that did not differ significantly from littermate controls given either vehicle or Exendin-4 (Figure 2Q left panel), and to result in restoration of the overall insulinemic response to control levels (Figure 2Q, right panel). We therefore next investigated potential mechanisms leading to reduced serum GLP-1 concentrations.

### 3.3 RT-SAKO mice have elevated renal DGKε and renal and plasma LPA, and LPAR1/3 antagonism restores GLP-1 and insulin levels and glucose tolerance

**Figure 3:**
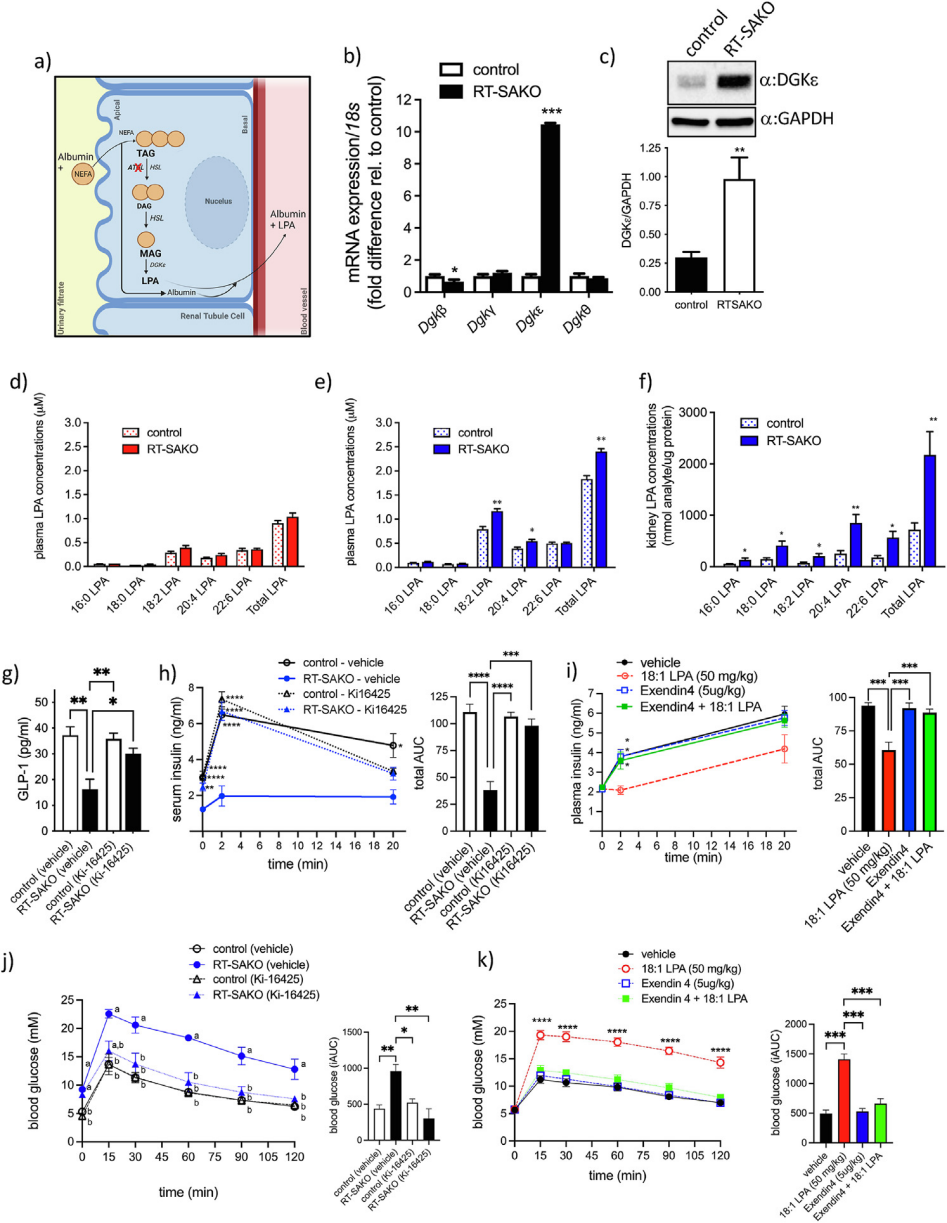
**Male RT-SAKO mice have elevated renal and plasma LPA, and LPAR1/3 antagonism restores GLP-1 levels, insulin levels and glucose tolerance.** (A) The postulated mechanism of increased LPA synthesis. Incoming non-esterified fatty acids (NEFA) carried on albumin are used to form triacylglycerol (TAG). HSL is expected to be the predominant TAG lipase in renal tubule cells deficient in *Atgl*, leading to increased production of monoacylglycerol (MAG) from diacylglycerol (DAG) that can be converted by DGKε-mediated phosphorylation to LPA, which is exported on albumin to the blood. (B) Male RT-SAKO mice (age 16–18 wks) have elevated kidney gene expression of *diacylglycerol kinase epsilon* (*Dgkε*). Data are means ± SEM, ∗P < 0.05, ∗∗∗P < 0.001 versus controls, (n = 6–9). (C) Immunodetectable levels of kidney DGKε were also elevated in male RT-SAKO mice compared to control littermates. Data are means ± SEM, ∗∗P < 0.01 (n = 6–7). Concentrations of total LPA and individual LPA species were analyzed in plasma collected from 16 to 18 wk old female (n = 3–4) (D) and male (n = 3) (E) RT-SAKO mice and their control littermates, and in lipid extracts from kidneys of male mice (n = 7) (F). Data are means ± SEM, ∗P < 0.05, ∗∗P < 0.01 versus littermate controls. (G) Treatment of mice with the LPAR1/3 antagonist Ki-16425 restored GLP-1 concentrations to littermate control levels in RT-SAKO mice but did not significantly affect GLP-1 levels in control mice. Data are means ± SEM, ∗P < 0.05, ∗∗P < 0.01, determined by 2-way ANOVA with Tukey's post-hoc test (n = 4). (H) GSIS in RT-SAKO mice was analyzed by measuring serum insulin 30 min after injection of mice with Ki-16425 (time 0), but immediately prior to glucose injection, and then at 2 min and 20 min after glucose injection. Data are means +/- SEM, ∗P<0.05, ∗∗P < 0.01, ∗∗∗∗P < 0.0001 versus other groups at the same timepoint as indicated, and as determined by 2-way ANOVA with Tukey's post-hoc test (n = 5) (left panel). Total AUC response is shown (right panel). Data are means +/- SEM, ∗∗∗P < 0.001, ∗∗∗∗P < 0.0001, 2-way ANOVA with Tukey's post-hoc test (n = 5). (I) GSIS in wildtype C57Bl/6J mice was measured 10 min after they were injected with LPA, with or without Exendin 4 pre-treatment (10 min before LPA administration) (left panel). Data are means ± SEM, ∗P < 0.05 versus LPA-treatment only at the same timepoint by 2-way ANOVA with Tukey's post-hoc test (n = 5). Total AUC response is shown (right panel). Data are means ± SEM, ∗∗∗P < 0.001 by 2-way ANOVA with Tukey's post-hoc test. (J) Male RT-SAKO mice were injected with Ki-16425 then subjected to glucose tolerance testing (left panel). ^ab^P<0.01 Groups with different superscript letters are significantly different at that timepoint. The overall blood glucose response was analyzed by iAUC analysis (right panel). Data are means ± SEM, ∗P < 0.05, ∗∗P < 0.01 as indicated and analyzed by 2-way ANOVA with Tukey's post-hoc test (n = 3–4). (K) Overnight fasted wildtype C57Bl/6J mice were injected with 18:1 LPA, with or without Exendin 4, immediately prior to glucose tolerance testing (left panel) (n = 5). ∗∗∗∗P < 0.0001 versus all other groups at that timepoint. Overall blood glucose response (iAUC) is shown in the right panel. Data are means ± SEM, ∗∗∗P < 0.001 by 2-way ANOVA with Tukey's post-hoc test (n = 5).

Since renal tubule TAG hydrolysis was specifically altered in RT-SAKO mice, we postulated that a blood-born metabolite derived from compensatory alterations in renal lipid metabolism may be involved in down-regulating GLP-1 and insulin levels, and impairing insulin secretion. We considered pathways that could be indirectly affected by alterations in TAG hydrolysis, with an emphasis on pathways that generate bioactive lipids implicated in glucose control. In the absence of ATGL, HSL is expected to function as the predominant cellular TAG hydrolase. While both ATGL and HSL can hydrolyze TAG, these enzymes differ in their specificity. ATGL is predominantly a TAG hydrolase, with little activity against DAG, while HSL has ∼10-fold greater specificity for diacylglycerol (DAG) than TAG [69]. If ATGL is deficient, and therefore HSL activity predominates in cellular lipolysis, the biochemical outcome of lipolysis is therefore expected to favor the production of monoacylglycerol (MAG) over DAG. We hypothesized that activation of this MAG by acylglycerol kinases [70] could lead to the increased generation of LPA species. LPA have a high affinity for albumin, and in the case of excessive production, would be expected to rise in concentration in the circulation. This postulated mechanism is illustrated in Figure 3A. Prior work by Rancoule et al. has shown that LPA can inhibit pancreatic insulin secretion [50], while we have recently reported that LPA directly inhibits L-cell GLP-1 secretion [71], making this bioactive lipid a plausible candidate for investigation.

Analysis of the expression of a series of diacylglycerol kinases (DGKs), which also have activity towards MAG [70], demonstrated a surprising 10-fold increase in the gene expression of one specific enzyme, DGKε (Figure 3B), which was associated with an ∼4-fold increase in total levels of the protein (Figure 3C). We analyzed concentrations of various LPA species in the plasma of female and male 16- to 18-wk-old RT-SAKO mice and their control littermates (Figure 3D,E). There were no significant differences between genotypes in LPA concentrations in plasma from 16- to 18-wk-old females (Figure 3D). However, male RT-SAKO mice had significantly higher total plasma LPA concentrations compared to their male control littermates, largely due to elevations in linoleoyl-LPA (18:2 LPA) and arachidonoyl-LPA (20:4 LPA) (Figure 3E). We investigated whether the kidneys could be a potential source of this plasma LPA and found that the total LPA content was over 2-fold higher in RT-SAKO male mice compared to littermate controls (Figure 3F). Linoleoyl-LPA levels in kidneys of male RT-SAKO mice were over 2.5-fold higher, while 20:4-LPA levels were over 3.3-fold higher than levels in control littermates (Figure 3F). In addition, other species of LPA were also elevated in the kidneys of male RT-SAKO mice, including palmitoyl-LPA (16:0-LPA; ∼2.4-fold higher), stearoyl-LPA (18:0-LPA; ∼2.8-fold higher), and docosahexaenoyl-LPA (22:6-LPA; ∼3.1-fold higher) (Figure 3F).

We investigated a possible causal role for the elevated plasma LPA observed in male RT-SAKO mice in mediating reduced GLP-1 and insulin levels, and glucose intolerance, using Ki-16425, an LPAR1/3 antagonist. As expected, serum GLP-1 levels in vehicle-treated male RT-SAKO mice were significantly lower than in vehicle-treated male littermate controls, but were restored to control levels in male RT-SAKO mice treated with Ki-16425 (Figure 3G). GLP-1 levels in control mice were not significantly affected by this antagonist (Figure 3G). Similarly, pre-treatment of overnight-fasted mice with Ki-16425 30 min prior to *i.p.* administration of glucose significantly restored basal insulin levels and the first-phase serum insulin response of RT-SAKO mice, as measured at 2 min (Figure 3H, left panel), with the total insulinemic response in this group over a 20 min period returning to vehicle-treated control levels (Figure 3H, right panel). This effect was recapitulated in wildtype C57Bl/6J mice, where injection prior to glucose administration with 18:1 LPA completely flattened the first-phase serum insulin response, but Exendin-4 co-treatment prevented this at a dose that did not further augment the insulinemic response when administered alone (Figure 3I, right and left panels). This restoration of the initial rise in insulin concentrations by Ki-16425 was associated with a dramatic correction of the glucose intolerance that was evident after glucose injection in RT-SAKO mice treated only with vehicle control (Figure 3J, left and right panels). Similarly, 18:1 LPA injection recapitulated this significant glucose intolerance in wildtype C57Bl/6J mice, but this was prevented by pre-treatment of mice with Exendin-4 (Figure 3K, left and right panels).

### 3.4 Islet measures and β-cell function are not significantly different between RT-SAKO and control mice

Our studies indicate that the effect of LPA in RT-SAKO mice is acute and reversible, since the restoration of GLP-1 levels (Figure 3G), GSIS (Figure 3H), and glucose tolerance (Figure 3J) by Ki-16425 in RT-SAKO mice is both rapid and complete. The restoration of GSIS by Exendin-4 in RT-SAKO mice (Figure 3I) also strongly implicates reduced GLP-1 secretion as a primary causal factor in LPA-mediated inhibition of insulinemia. However, Rancoule et al. have previously reported effects of chronic modulation of LPA signaling on pancreatic islet number, suggesting a direct role of LPA on islet health may also be a factor [50]. We therefore tested isolated β-cell function and analyzed a series of pancreatic and islet measures.

**Figure 4:**
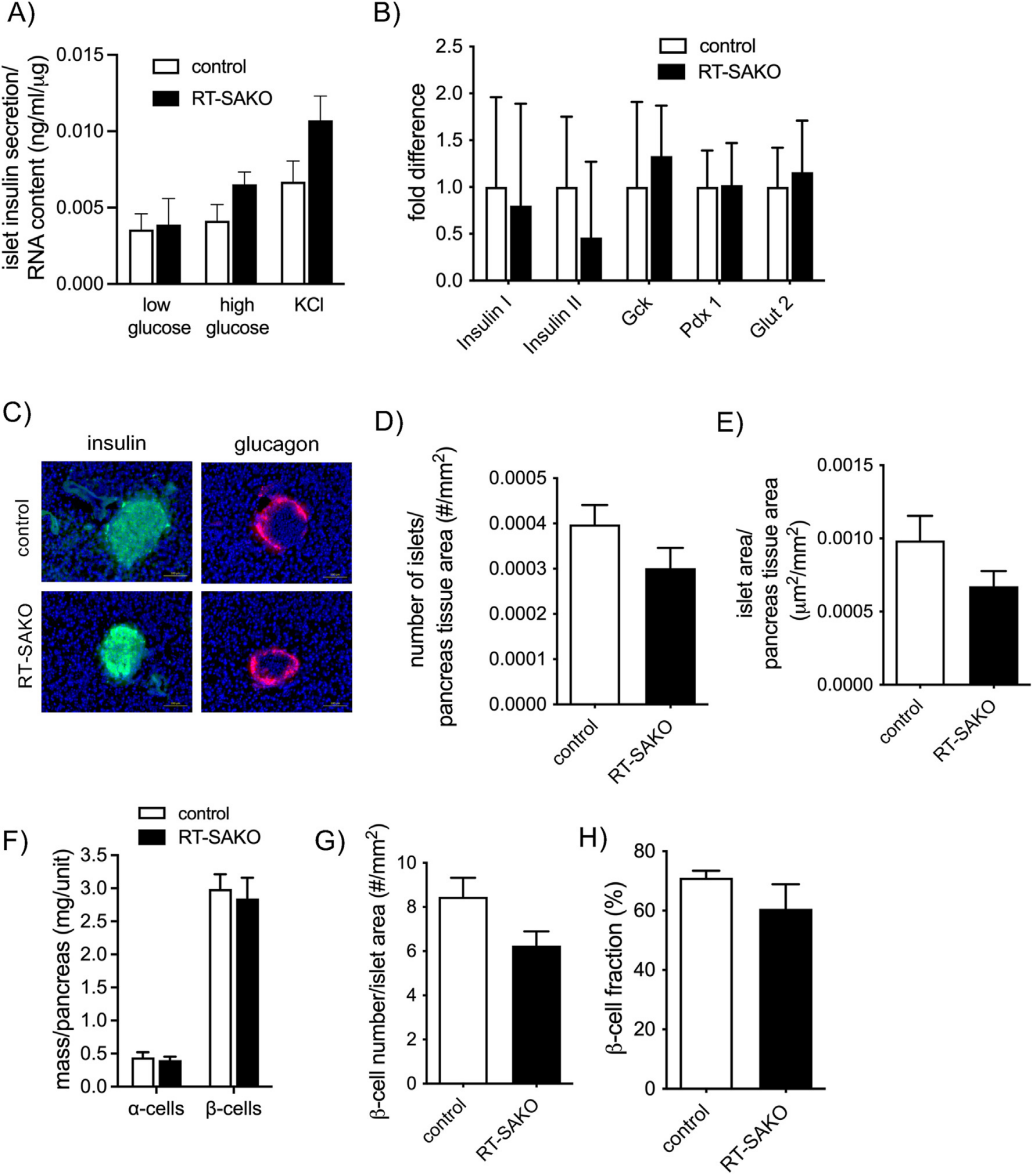
**Islet measures and β-cell function do not differ significantly between 16**–**18 wk old male RT-SAKO and control mice.** (A) Islets isolated from RT-SAKO mice and wildtype littermates exhibited similar levels of insulin secretion under various conditions (n = 5–6). (B) Pancreatic expression of genes involved in glucose and insulin metabolism (i.e., *Insulin I*, *Insulin II*, *glucokinase* (*Gck*), *pancreatic and duodenal homeobox 1* (*Pdx1*), and *glucose transporter 2* (*Glut2*) did not differ significantly between control and RT-SAKO littermates (n = 10). (C) Representative images of immunohistologically stained sections of pancreas from control and RT-SAKO mice identifying α-cells (identified in red by detection of glucagon) and β-cells (identified in green by detection of insulin). (D) Quantification of the number of islets per mm^2^ of cross-sectional pancreatic tissue analyzed (n = 5). (E) Islet cross-sectional area in μm^2^ per mm^2^ of pancreatic tissue (n = 5). (F) Mass of α-cells and β-cells per pancreas (n = 12). (G) Number of β-cells per islet cross-sectional area (n = 5) and (H) β-cells as a fraction of islet cells (percent) (n = 5). All data shown are means ± SEM.

Islets isolated from RT-SAKO and control littermate mice were washed and incubated for 2 h prior to use in testing, in order to wash out LPA from the host animal's environment and allow for observation of existent function. When incubated in low- or high-glucose conditions, or when treated with KCl, which acts to non-specifically depolarize the plasma membrane to elicit insulin secretion, isolated islets secreted insulin at levels that did not differ significantly between the genotypes (Figure 4A). Importantly, no impairment in insulin secretion was evident in islets isolated from RT-SAKO mice that could explain the impaired *in vivo* GSIS and glucose intolerance in these mice (Figure 4A). The expression of genes involved in insulin biosynthesis (*Insulin I* and *Insulin II*), glucose sensing (*Gck, Glut2*), β-cell maturation (*Pdx1*), and glucose uptake and sensing (*Glut2*) in islets isolated from RT-SAKO and control littermates did not differ significantly (Figure 4B). Islet morphology was visualized using antibodies for insulin and glucagon (Figure 4C). The number of islets per cross-sectional area of pancreas (Figure 4D), and the area occupied by islets per cross-sectional area of pancreas (Figure 4E) did not differ significantly between RT-SAKO and control littermates. The total mass of β-cells per pancreas did not differ significantly between RT-SAKO and control mice, nor did the total mass of α-cells (Figure 4F), and no significant differences were detected between genotypes in the number of β-cells per islet area (Figure 4G), or the β-cell fraction per islet (Figure 4H).

## 4 Discussion

Chronic, low grade inflammation is implicated in the development of T2DM [72,73], since it worsens insulin resistance and contributes directly to impaired GSIS [[74], [75], [76]]. Low-grade inflammation also occurs in conditions that act as major risk factors for this disease, including obesity and metabolic syndrome (reviewed in [77]). Several mechanisms have been identified that help to explain these associations, but the links between obesity, inflammation, and T2DM are not fully understood.

In the current study, we observed that impaired glucose tolerance develops in RT-SAKO mice in an age- and sex-dependent manner, such that this effect was evident in 16- to 18-week-old male mice, but not in female littermates, or in younger (*i.e.,* 10- to 12-week-old) mice of either sex. We initially hypothesized that this effect would be the result of inflammation-induced insulin resistance, resulting from kidney lipotoxicity due to the progressive accumulation of TAG in renal tubule cells. However, insulin tolerance testing and HIEC indicated normal insulin sensitivity at the whole organism and hepatic levels, while serum creatine levels in 16- to 18-week old male mice did not differ between the genotypes, suggesting comparable kidney health. The absence of a renal inflammatory or lipotoxic phenotype was supported by analyses indicating no differences in kidney expression of genes involved in inflammation, fibrosis, or repair. It was therefore necessary to consider alternative hypotheses.

Analysis of major blood lipid species did not identify any differences between genotypes in male mice. A broader search for differences in blood hormonal and metabolic regulators between genotypes occurring in 16- to 18-week-old male, but not female mice, indicated that most factors (*i.e.,* glucagon, GIP, leptin, and resistin) also did not offer insight. However, two specific differences emerged. Serum concentrations of both insulin and GLP-1 were significantly lower in male RT-SAKO mice compared to littermate controls, while these genotype-dependent differences were not apparent in age-matched female mice that maintained a normoglycemic response to glucose administration. The response of β-cells to a rise in blood glucose is modulated by the action of gut-derived incretin hormones, including GLP-1 that potentiates GSIS [78], and is particularly important for the first phase of GSIS [79,80]. In this regard, an impaired first phase insulin secretory response was indeed evident in male RT-SAKO mice at this age and was restored by treatment of mice with the GLP-1 receptor agonist Exendin 4, supporting the notion that a specific GLP-1 deficiency was a causal factor in impaired GSIS, reduced circulating insulin concentrations, and impaired glucose tolerance in these mice. We therefore next began a search for underlying mechanisms.

The major hormonal changes in this model were decreased circulating GLP-1 and insulin. These factors are secreted by cells that are located at sites distal to the kidneys, yet given the nature of the model, the signal for these changes likely originated from altered renal lipid metabolism. We therefore postulated that a blood-borne bioactive lipid metabolite of kidney origin was likely involved. In cells deficient in ATGL, HSL is expected to be the predominant TAG lipase [81]. This enzyme does not fully compensate for ATGL, since mice deficient in *Atgl* have elevated TAG in most tissues, including the kidneys, although TAG breakdown does proceed [82]. However, the greater specificity of HSL for DAG over TAG would be predicted to favour formation of monoacylglycerol [83]. We analyzed the expression of major kidney lipid kinases, and found a 10-fold increase in renal gene expression of one lipid kinase, *Dgkε*, in 16- to 18-week-old male RT-SAKO mice. This was accompanied by an ∼4-fold higher level of the protein.

DGKε can use either 1- or 2-monoacylglycerol to generate LPA species [70] and, indeed, the total LPA content in kidneys from 16 to 18 week old male RT-SAKO mice was found to more than double, while contents of all individual species examined also increased significantly. Notably, a rise in blood LPA total and species-specific levels was evident in 16- to 18-week-old male RT-SAKO mice, but not in females. Lysophosphatidic acid generated in renal tubule cells could be exported with albumin, potentially as a mechanism to dissipate the excess lipids resulting from impaired TAG breakdown, and this would also be expected to alter signaling at distal sites [84]. Indeed, a similar mechanism has been implicated in the export of LPA from cancer cells [85]. These bioactive lipids are implicated in potent biological signaling effects throughout the body and, importantly, we have recently reported that various species of LPA can inhibit the secretion of GLP-1 by enteroendocrine L-cells, and reduce blood GLP-1 levels in a manner that is dependent on LPA receptor 1/3-mediated signaling [71]. In the current work, blockade of LPAR1/3 by Ki-16425 restored blood GLP-1 to control levels, corrected the impairment in first phase GSIS, and restored glucose tolerance in 16- to 18-week-old male RT-SAKO mice, indicating a causal role for LPA in these metabolic alterations. Of note, the current study did not temporally monitor blood GLP-1 levels during GSIS testing or drug rescue tests, which is a limitation of the work. Future studies should measure blood glucose, insulin, and GLP-1 levels concomitantly over time to provide additional insight into their coordinate regulation following LPAR antagonism. However, to the best of our knowledge, the current study is the first to demonstrate a direct role for endogenously derived LPA in the regulation of GLP-1 levels.

This study is also the first to implicate renal DGKε in blood LPA production. Although there are multiple pathways and sites for LPA synthesis in the body, in the absence of inflammation Autotaxin, a lysophospholipase D found both in tissues and in circulation [86], is typically considered the major regulator of blood LPA concentrations [87]. It is normally responsible for ∼40 % of total circulating LPA [86], but can be upregulated in obesity in both mice and humans to increase LPA generation [88]. However, neither adipose tissue mass, nor kidney *Autotaxin* expression were altered in RT-SAKO mice in the current study, and we did not detect a rise in renal markers of inflammation. The current work therefore suggests that organs and enzymes, such as kidney DGKε, which are not typically thought to contribute significantly to circulating LPA may, indeed, be significant sources under certain conditions, and future studies should consider alternate pathways and sites for LPA generation in metabolic disease. Additionally, given that loss of DGKε is known to cause an atypical hemolytic-uremic syndrome [89], future work should also investigate whether synthesis of LPA by this enzyme is mechanistically implicated.

LPA concentrations are reported to be increased in the blood in inflammation and chronic disease states [87], and in obesity [90]. Although, in the current model, LPA appeared to be derived from altered renal lipid metabolism rather than a pro-inflammatory or obesity-related cascade, this finding nonetheless has relevance for understanding links between inflammation and glycemic regulation. Notably, inflammation, chronic disease states, and obesity, are all conditions where reduced GLP-1 levels and action are also often observed [91]. Identifying factors that link impaired GLP-1 secretion to obesity and inflammation can help to generate a better understanding of why the comorbid occurrence of these conditions contributes to progressive worsening of T2DM and other related diseases, and may be key to disrupting vicious cycles between these diseases. This understanding has potentially broad implications. While the health benefits of GLP-1 activity in blood glucose lowering are well established [92], GLP-1 activity is also implicated as a protective factor in a host of other diseases including myocardial ischemic injury [[93], [94], [95], [96], [97]] and various dementias [[98], [99], [100], [101]]. Strategies to prevent decline in GLP-1, and/or raise endogenous GLP-1 levels to an effective range, are thus likely to have clinical benefits beyond improving blood glucose regulation.

Rancoule et al. [50] have previously found that antagonism of LPA-mediated signaling can improve insulinemia and glycemic control in high-fat diet-fed obese mice with elevated circulating LPA [50]. In that study, a direct role for LPA in the inhibition of insulin secretion from isolated islets was also found. The current study, which indicates a role for endogenously derived LPA in the modulation of the incretin hormone GLP-1, sheds further light on that work. In addition to potentiating GSIS, GLP-1 can help to promote the survival of β-cells [102]. In Rancoule's study, chronic administration of Ki-16425 significantly increased islet number in high-fat diet-fed mice, although most other measures of islet and β-cell health were unaltered. While this may have resulted from direct antagonism of the detrimental effects of LPA on β-cells, our work suggests that it may also reflect the action of anticipated improvements in GLP-1 concentrations in this model following antagonism of LPA-mediated suppression of GLP-1 secretion. Likewise, the findings of Rancoule et al. [50] suggest that in the current study, the counteraction of LPA signaling in islets of RT-SAKO mice may have contributed to improved GSIS and glucose tolerance following acute Ki-16425 administration. However, a direct role for GLP-1 in these effects is also supported by the restoration of GLP-1 levels through Ki-16425 administration, and the restoration of first phase insulin secretion by Exendin 4. Notably, in the current study, no significant differences in β-cell or islet health or function were detected, despite extensive evaluation. Future studies on conditions with altered circulating LPA levels should therefore examine roles for both GLP-1 and β-cell function in mediating outcomes related to glycemic control.

The importance of generating an organ-specific, and even a cell-type specific model to examine effects of loss of *Atgl* is highlighted by the profound differences in kidney health between RT-SAKO mice and mice globally deficient in this enzyme. *Atgl* total knockout mice at only 9–10 weeks of age have renal TAG over-storage and evidence of lipotoxicity, including increased podocyte apoptosis and damage to the glomerular filtration barrier, along with smaller kidneys [36] and marked proximal tubule damage [37]. Conversely, we did not find evidence of kidney lipotoxicity, impaired function, cellular damage, or increased repair, despite increased TAG storage in renal tubule cells of the RT-SAKO mice. Renal tubule cells are susceptible to lipotoxic injury in a variety of models [103], and blood LPA concentrations can be a predictor of kidney injury and disease development [104,105]. The unexpected absence of renal tubule damage may reflect the novel and specific capacity of cells in this model to dissipate lipotoxic stress. In this regard, our finding of elevated LPA in kidneys and in blood raises the intriguing possibility that the conversion and export of metabolites of TAG hydrolysis may constitute a novel mechanism whereby renal tubule cells act to avoid the accumulation of lipotoxic intermediates. Alternately, it suggests that other factors present in the *Atgl* global knockout, but absent from the RT-SAKO targeted model, may be critical for the development of renal lipotoxicity. Mice globally deficient in ATGL activity develop obesity, widespread ectopic lipid accumulation, and insulin resistance [82]. These characteristics were notably absent from the RT-SAKO model.

Future study in humans should examine differences between sexes. This model demonstrates an interesting sexual dimorphism in the development of dysglycemia that is reflected in human disease. Advancing age is the greatest risk factor for the most prevalent diseases of developed countries [106] and, overall, CKD [107] and T2DM [108] tend to occur at a younger age in men than women, who often ‘catch-up’ in relative incidence during the post-menopausal years. It is notable that blood LPA concentrations increase in both male and female rats [109] and humans [90] with advancing age, and plasma LPA concentrations are significantly higher in women than men [90]. This suggests women may have a different response to LPA, or a better capacity to metabolize and tolerate LPA, but that this ability is attenuated with the decline in female sex hormone levels that occurs following menopause. Indeed, there is evidence of a role for sex hormone-mediated signaling acting as a modifier in the action of LPA [[110], [111], [112]]. Although the nature of this role is only beginning to be understood, it is plausible that sex differences in LPA signaling and metabolism are a factor in differential chronic disease development.

Overall, the RT-SAKO mouse model presents an opportunity to study new mechanistic links, and challenge prior notions on the nature of established links between obesity, CKD, and DM. Kidney lipid droplet accumulation is common in all three conditions, and this model allows for the study of metabolic and pathophysiological changes downstream of renal steatosis and, therefore, assessment of a causal role. Blood LPA is also elevated in human obesity [113,114], in patients with CKD [115], and in aging [90,109], and is functionally implicated in the progression of diabetic nephropathy [116] – conditions that are all associated with renal steatosis. Whether human kidney is a source of elevated LPA in those conditions is currently unknown. However, it is reasonable to believe that whenever selective insulin resistance is present in renal tubules, an elevation in HSL activity relative to ATGL activity could result in the promotion of MAG formation, and subsequently increased LPA biosynthesis [66]. Insulin is a powerful negative regulator of stimulated, HSL-mediated lipolysis, since it activates phosphodiesterases that degrade cAMP, the second messenger responsible for activating protein kinase A to phosphorylate HSL and cause its translocation to lipid droplets [83]. In primary proximal tubule cells, insulin resistance has been found to impair the inhibitory role of insulin on cAMP-mediated processes related to glucose metabolism [66], and it makes sense that this would extend to loss of inhibition of lipolysis as well. Given associations identified in the current work, further study of the regulation of lipolysis in human kidneys from patients with obesity, CKD and DM is merited.

## Funding

This work was supported by grants to 10.13039/100011115R.E.D. from Diabetes Canada (10.13039/100007476Canadian Diabetes Association Grant Number NOD_OG-3-15-4933-RD), Canada Foundation for Innovation—Leader's Opportunity Fund and Government of Ontario–Ontario Research Fund (Project No. 30259), Government of Ontario Early Researcher Award (ERA)—Ministry of Research, Innovation and Science (ER16-12-162), Natural Sciences and Engineering Research Council of Canada (NSERC) #RGPIN-2019-05642, #RGPIN-2012-418213, and RGPAS-2019-00008, and the Waterloo Commercialization Office (WatCo) Women in STEM-Prototype Development/Demonstration Project Award (Project—WatCo Ref 10173); by a grant to J.W.J. from the Canadian Institutes of Health Research (CIHR) #PJT-159552; and by grants to K.D.S. from NSERC #RGPIN-2018-04334, an infrastructure grant from the Canada Foundation for Innovation–Innovation Fund and Government of Ontario–Ontario Research Fund (Project No. 33433). Salary support was provided from the Canada Research Chairs program for K.D.S. who was a Chair in Nutritional Lipidomics (950–228125). J.J.A.-H. was supported by an NSERC Doctoral Scholarship (PGS-D), an Ontario Graduate Scholarship, and the University of Waterloo President's Scholarship. Clamp studies were supported by a grant from the Canadian Institutes of Health Research (#FDN-143247) to A.M. who was the holder of a Pfizer/CIHR partner Chair in the pathogenesis of insulin resistance and cardiovascular diseases.

The funding agencies had no role in the study design, the collection, analysis or interpretation of data, the writing of the report, or the decision to submit the article for publication.

## CRediT authorship contribution statement

**Maria F. Fernandes:** Data curation, Formal analysis, Investigation, Methodology, Supervision, Writing – original draft, Writing – review & editing. **Juan J. Aristizabal-Henao:** Data curation, Formal analysis, Investigation, Methodology, Writing – original draft. **Phillip M. Marvyn:** Data curation, Formal analysis, Investigation, Writing – original draft. **Iman M'Hiri:** Data curation, Formal analysis, Investigation, Methodology, Writing – original draft. **Meghan A. Wiens:** Data curation, Formal analysis, Investigation. **Monica Hoang:** Data curation, Formal analysis, Investigation. **Manuel Sebastian:** Data curation, Formal analysis, Investigation. **Renato Nachbar:** Data curation, Formal analysis, Investigation, Writing – original draft, Writing – review & editing. **Philippe St-Pierre:** Data curation, Formal analysis, Investigation. **Kalsha Diaguarachchige DeSilva:** Data curation, Formal analysis, Investigation. **Geoffrey A. Wood:** Formal analysis, Investigation. **Jamie W. Joseph:** Conceptualization, Formal analysis, Funding acquisition, Supervision. **Christine A. Doucette:** Conceptualization, Formal analysis, Funding acquisition, Supervision, Writing – review & editing. **André Marette:** Conceptualization, Formal analysis, Funding acquisition, Supervision, Writing – review & editing. **Ken D. Stark:** Conceptualization, Formal analysis, Funding acquisition, Supervision. **Robin E. Duncan:** Conceptualization, Data curation, Formal analysis, Funding acquisition, Investigation, Methodology, Project administration, Supervision, Writing – original draft, Writing – review & editing.

## Declaration of competing interest

The University of Waterloo Commercialization Office (WatCo) has applied for WIPO (PCT) Patent Application No. CA2021/051536, Titled: Modulation of glucagon-like peptide 1 and uses thereof (filed 29 October 2021) on behalf of the inventor (R.E.D.), and M.F.F., J.J.A.-H. and K.D.S, are included in a revenue-sharing agreement if commercial application is developed. This patent application includes all data reported in the current manuscript on GLP-1 levels.

## Data Availability

Data will be made available on request.
