# Supplementary material for: Renal tubule-specific Atgl deletion links kidney lipid metabolism to glucagon-like peptide 1 and insulin secretion independent of renal inflammation or lipotoxicity
Source: Mol Metab. 2024 Jan 26;81:101887. doi: 10.1016/j.molmet.2024.101887 (PMC10850971; doi:10.1016/j.molmet.2024.101887)
Supplement: Multimedia component 1 [file mmc1.pdf]

Supplementary Figures and Methods

Renal tubule-specific *Atgl* deletion links kidney lipid metabolism to glucagon-like peptide 1 and insulin secretion independent of renal inflammation or lipotoxicity

Maria F. Fernandes[1], Juan J. Aristizabal-Henao[1], Phillip M. Marvyn[1], Iman M'Hiri[1], Meghan A. Wiens[1], Monica Hoang[2], Manuel Sebastian[3], Renato Nachbar[4], Philippe St-Pierre[4], Kalsha Diaguarachchige DeSilva[1], Geoffrey A. Wood[5], Jamie W. Joseph[2], Christine A. Doucette[3], André Marette[4], Ken D. Stark[1], and Robin E. Duncan*[1]

[1]Department of Kinesiology and Health Sciences, University of Waterloo, Canada [2]School of Pharmacy, University of Waterloo, Canada [3]Max Rady College of Medicine, University of Manitoba, Canada [4]Quebec Heart and Lung Institute, Department of Medicine, Laval University, Québec, Canada [5]Ontario Veterinary College, University of Guelph, Canada

*Corresponding Author. Department of Kinesiology and Health Sciences, University of Waterloo, 200 University Ave W, Waterloo, ON, Canada, N2L 3G1. E-mail: reduncan@uwaterloo.ca

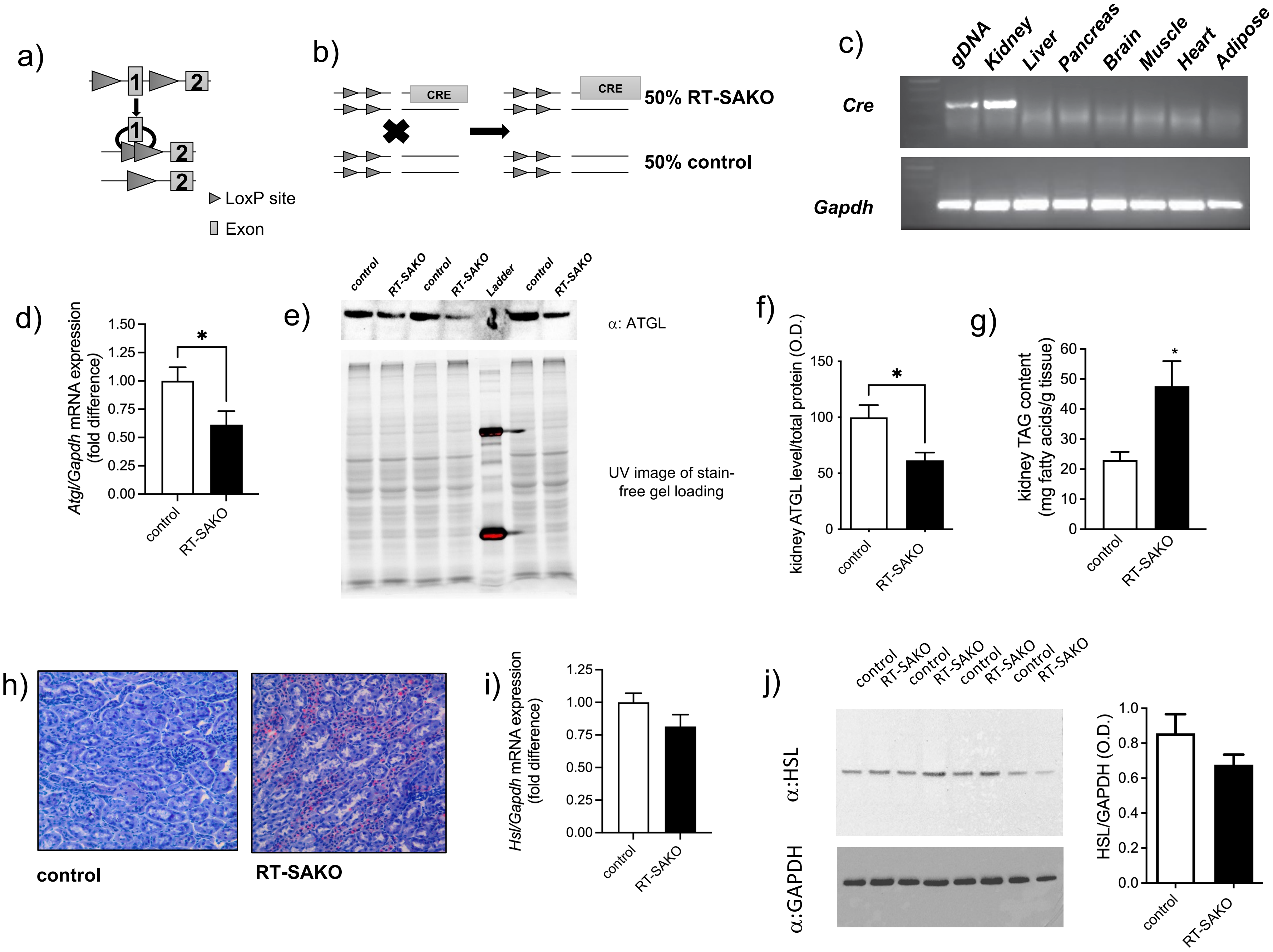

Supplementary Figure 1. (a) The entire first exon of *Atgl* containing catalytically critical residues was flanked by LoxP sites in *Atgl flox/flox* mice, resulting in excision in the presence of *Cre recombinase.* (b) Mice were generated by crossing *Ksp1.3/Cre* transgenic mice (B6.Cg-Tg(Cdh16-cre)91Igr/J) with *Atgl flox/flox* mice to generate mice heterozygous for *Cre* and for the floxed *Atgl* allele, which were backcrossed again with *Atgl flox/flox* mice to generate mice homozygous for the floxed *Atgl* allele, and heterozygous for *Cre.* These mice, illustrated in the top left part of (b) were routinely crossed with *Atgl flox/flox* mice (illustrated in the bottom left part of (b)) to generate 50% RT-SAKO mice and 50% control littermates. (c) Mice were routinely genotyped by PCR amplificaton of *Cre recombinase* in genomic DNA (gDNA) isolated from mice. Notably, RNA expressing *Cre recombinase* was detected in kidneys but absent in all other tissues tested by RT-PCR, highlighting the specificity of the model. A representative blot is shown. (d) Kidney expression of *Atgl* was normalized to *Gapdh* and expressed relative to control values (n=8-9). Data are means ± SEM, *P<0.05. (e) A representative immunoblot of ATGL in kidney lysates is shown (top) with an image of total protein loading (bottom) determined by UV illumination of the TGX Stain-free gel (n=3). (f) Quantification of the optical density (O.D.) of immunodetectable kidney ATGL normalized to total protein per lane and expressed as a percentage of control (n=3). Data are means ± SEM, *P<0.05. (g) Kidney TAG content was analyzed (n=7). Data are means ± SEM, *P<0.05. (h) Kidney cryosections were stained for lipids using Oil Red O, and counterstained with hematoxylin and eosin to show cellular structures. Representative images are shown. (i) Kidney expression of *Hsl* was normalized to *Gapdh* and expressed relative to control values. Data are means ± SEM, (n=8-9). (j) A representative immunoblot of HSL and GAPDH protein levels is shown (left panel) with quantification (right panel). Data are means ± SEM, (n=6).

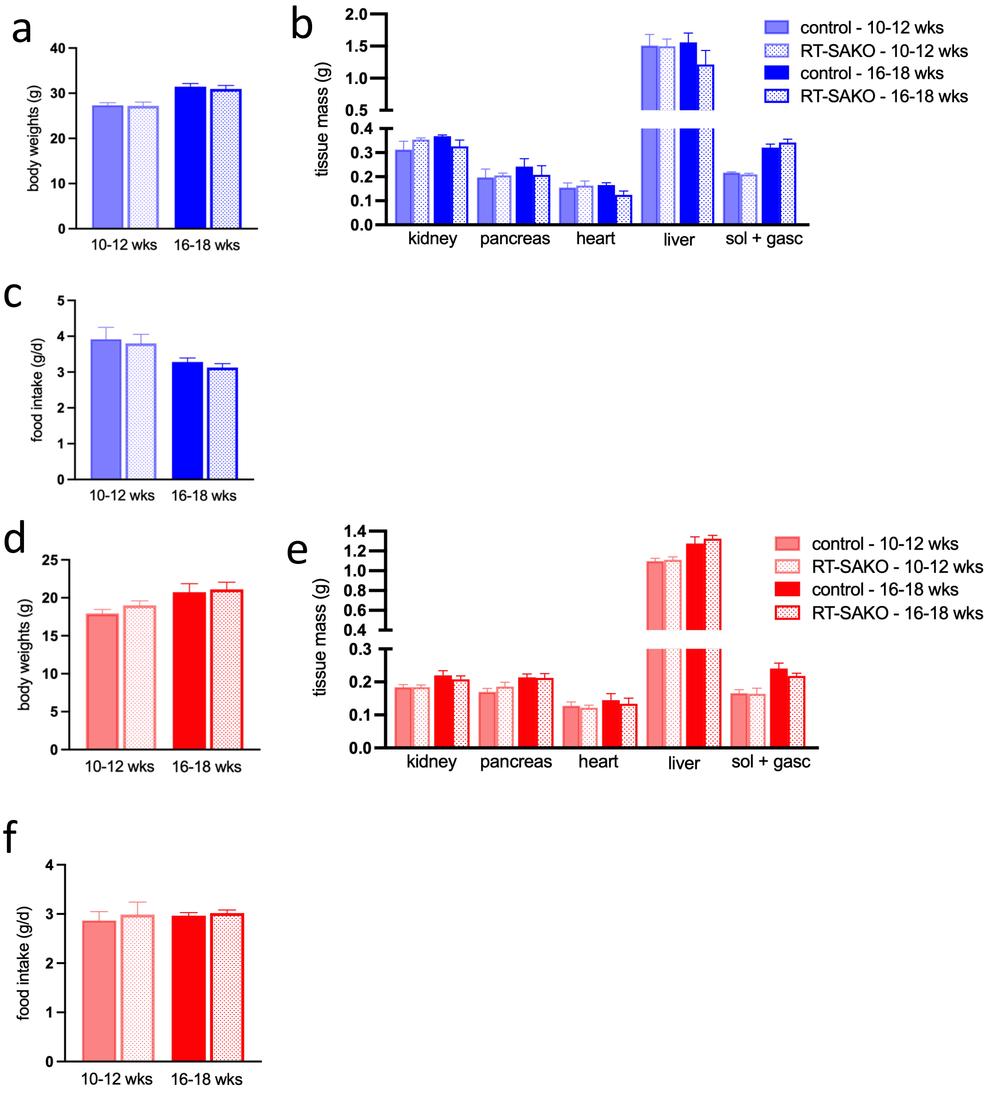

Supplementary Figure 2. Body weights, tissue masses and food intakes of male and female RT-SAKO mice and control littermates at 10-12 wks and 16-18 wks of age. Male RT-SAKO and control littermate body weights (n=10-12) (a), tissue masses at necropsy (n=3-7) (b), and daily food intakes (n=4-10) (c) were recorded at 10-12 and 16-18 wks of age. Female RT-SAKO and control littermate body weights (n=5) (d), tissue masses at necropsy (n=5-6) (e), and daily food intakes (n=5) (f) at 10-12 and 16-18 wks of age.

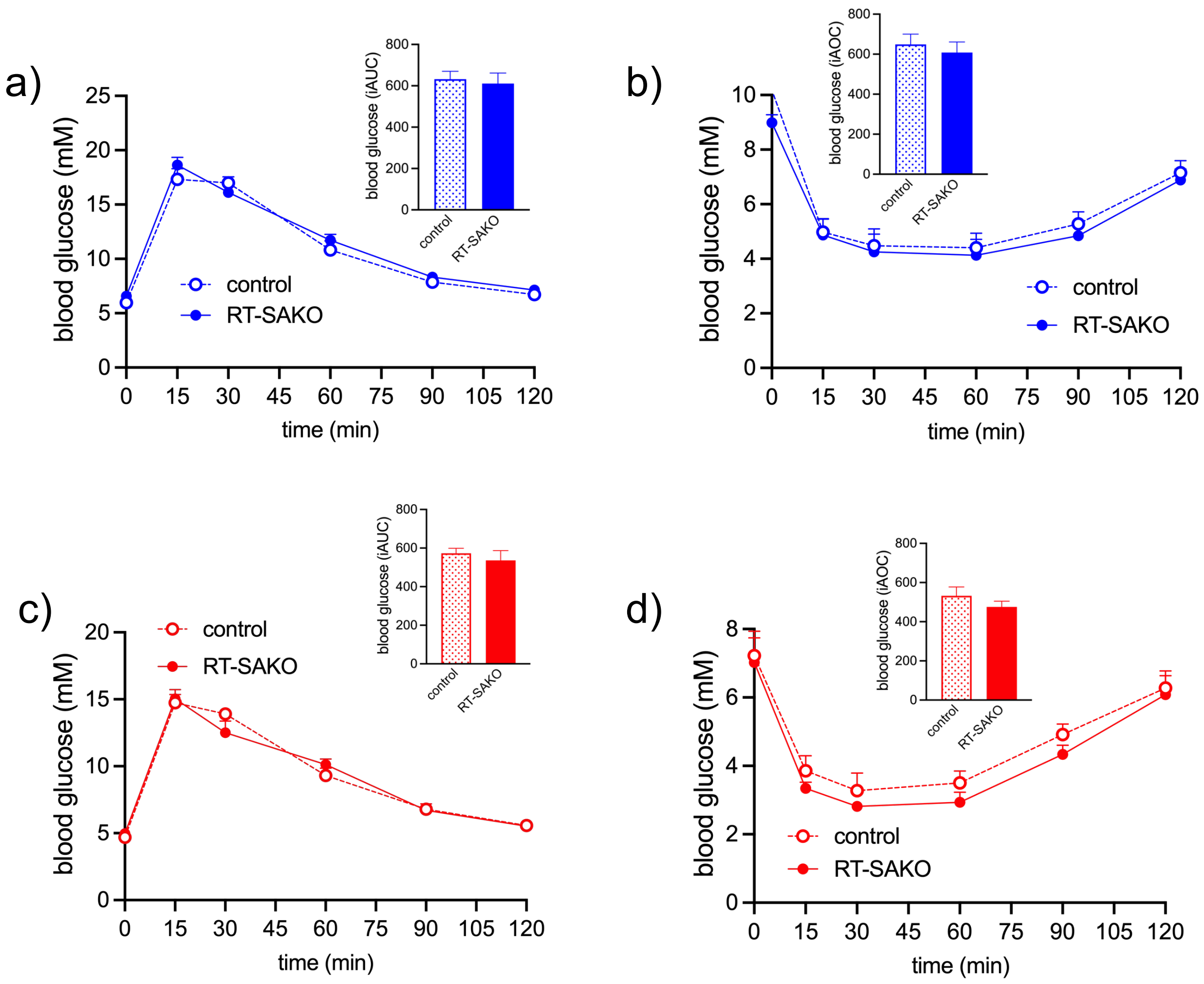

Supplementary Figure 3. Glucose tolerance and insulin sensitivity in male and female RT-SAKO mice and their control littermates at 10-12 weeks of age. Male RT-SAKO mice and their control littermates exhibit similar glucose tolerance (n= 5-7) (a) and insulin sensitivity (n=4-7) (b) at all timepoints during a 2 hr test, and overall when net incremental area-under-the-curve (iAUC) and net incremental area-over-the-curve (iAOC) are calculated (insets to figures). Female RT-SAKO mice and their control littermates have similar glucose tolerance (n=7) (c) and insulin sensitivity (n=5) (d) during a 2 hr test, and when net iAUC and net iAOC are calculated (insets to figures).

Supplementary Methods

S1. Generation of RT-SAKO mice. We used the Cre-LoxP-recombination system to excise the catalytic region of *Atgl (Pnpla2)* to generate the renal tubule-specific ATGL knockout mice (RT-SAKO) and littermate controls using a strategy illustrated in Supplementary Figure 1a and b. The *Atgl* flox/flox mice were generously donated by Dr. Hei Sook Sul and have been described previously [1, 2]. These mice have the *LoxP* sites flanking the first exon of *Atgl,* which contains the serine/aspartate catalytic dyad. ATGL flox/flox mice were backcrossed for ≥ 12 generations onto a C57Bl/6J (The Jackson Laboratory, JAX stock #000664) background strain, and mated with Ksp1.3/Cre transgenic mice (B6.Cg-Tg(Cdh16-cre)91Igr/J), which express Cre recombinase under control of the cadherin 16 promoter (The Jackson Laboratory, JAX stock #012237). Ksp1.3/Cre transgenic mice express Cre-recombinase primarily in renal tubule cells and glomeruli [3], and have been used previously to generate other renal tubule-specific knockout mouse models [4, 5]. The resulting offspring were genotyped for the presence of *Cre* and for heterozygosity of the *Atgl* floxed allele, and were then backcrossed with mice lacking Cre but homozygous for the Atgl floxed allele, to generate mice homozygous for the Atgl floxed allele and heterozygous for *Cre*. Genotyping for presence of the *LoxP* flanked allele and loss of the first exon after *Cre*-mediated recombination was performed as previously described [1, 2]. RT-SAKO and control mice were generated by mating *Atgl* flox/flox mice heterozygous for *Cre* (*i.e.* RT-SAKO mice) with *Atgl* flox/flox mice wildtype for *Cre*, generating 50% RT-SAKO and 50% littermate control mice.

S2. Analysis of the Cre transgene. Mice were genotyped for the presence of *Cre recombinase*, which resulted in the generation of a 270 bp amplicon in mice carrying the transgene. Expression of *Cre recombinase* in a variety of tissues was assessed by PCR following cDNA generation from extracted mRNA, and a representative RT-PCR blot is shown in Supplementary Figure 1c. Primers for the amplification of *Cre* in tissues and genomic DNA are provided in the primer table in S3.

S3. PCR Primer Sequences.

Primer Sequences

Gene	Direction	Sequence
Atgl-loxP	forward	5'-ctcaccgccacagcgctggtcac-3'
	reverse	5'-gtccctctctaccgcttctac-3'
Ksp-cre	forward	5'-aggttcgttcactcatgga-3'
	reverse	5'-tcgaccagtttagttaccc-3'
Insulin I	forward	5' - gctggtagagggagcagatg - 3'
	reverse	5' - cagagaccatcagcaagcag - 3'
Insulin II	forward	5'-atggccctgtggatccgctt-3'
	reverse	5'-ctagttgcagtagttctcca-3'
Gsk	forward	5'-cgtggatggctccgtgtac-3'
	reverse	5'-ggcgtgaaaccgctcctt-3'

Pdx1	forward	5'-ggcgtcgcacaagaagaaa-3'
	reverse	5'-tctctccggctatacccaactg-3'
Glut2	forward	5'-tggaaggatcaaagcaatgttg-3'
	reverse	5'-catcaagagggctccagtcaa-3'
Atgl	forward	5’-aacgccactcacatctacgg-3’
	reverse	5’-gcctccttggacacctcaata-3’
Hsl	forward	5’-ggagtctatgcgcaggagtg-3’
	reverse	5’-gcttcttcaaggtatctgtgcc-3’
Ki67	forward	5’-ctgcctgcgaagagagcatc-3’
	reverse	5’-agctccacttcgccttttgg-3’
Pcna	forward	5′-tgctctgaggtacctgaact-3′
	reverse	5′-tgttcctcatcttcaatct-3′
Tnf-α	forward	5’-caacgccctcctggccaacg-3’
	reverse	5'-tcggggcagccttgtccctt-3'
Il1β	forward	5'-cctgctggtgtgtgacgttccc-3'
	reverse	5'-gggtccgacagcacgaggct-3'
Il6	forward	5'-gctggagtcacagaaggagtggct-3'
	reverse	5'-ggcataacgcactaggtttgccgag-3'
Tgfβ1	forward	5’-tgagtggctgtcttttgacg-3’
	reverse	5'-gtggtaggtactgtacttgg-3'
Col1a1	forward	5’-gaaacccgaggtatgcttga-3’
	reverse	5'-tacatcctcagctccctggg-3'
Col4a1	forward	5’-ctggagaaaagggccagat-3’
	reverse	5'-acctgtccgtgttcaattcct-3'
Fn	forward	5'-acatggctttaggcggacaa-3'
	reverse	5'-ttcggcaggtatggtcttgg-3'
Cd36	forward	5'-tcctctgacatttgcaggtccatct-3'
	reverse	5'-aggcaaaggcgttggctggaa-3'
Ctgt	forward	5’-agcagctgggagaactgtgt-3’
	reverse	5′-gtttcgtcgacgtttatggt-3′
Dgat1	forward	5’-ctggattgtgggccgattct-3’
	reverse	5’-atacatgagcacagccaccg-3’
Dgat2	forward	5’-aagacatcgacctgtaccatgc-3’
	reverse	5’-ctcagtctctggaaggccaaa-3’
Fabp4	forward	5’-gtgggatggaaagtcgacca-3’
	reverse	5’-cataacacattccaccaccagc-3’
Fas	forward	5’- ttgctggcactacagaatgc -3’
	reverse	5’- aacagcctcagagcgacaat -3’
Lipin1	forward	5’-ccttagggagccggaagact-3’

	reverse	5'-attgttggcgactggtcact-3'
G6pc	forward	5'-cagtggtcggagactggttc-3'
	reverse	5'-tataggcacggagctgttgc-3'
Gck	forward	5'-caactggaccaagggcttcaa-3'
	reverse	5'-tgtggccaccgtgtcattc-3'
Hk1	forward	5'-gtgggatggaaagtcgacca-3'
	reverse	5'-cataacacattccaccaccagc-3'
Hk2	forward	5'-actgtggctaaatgagactggg-3'
	reverse	5'-accatgccaaggagcttgat-3'
Hk3	forward	5'-cacttaaccaatctcggagt-3'
	reverse	5'-aggctatcactttcgatctc-3'
Sglt1	forward	5'-ctgcccatgttcctcatggt-3'
	reverse	5'-tggtgtgccgcagtatttct-3'
Sglt2	forward	5'-ttgtgttggcttgtggtct-3'
	reverse	5'-atgttgctggcgaacagaga-3'
Enpp2	forward	5'-gaccctaaagccattattgctaa-3'
	reverse	5'-gggaaggtgctgtttcatgt-3'
Kim-1	forward	5'-acatatcgtggaatcacaacgac-3'
	reverse	5'-acaagcagaagatgggcattg-3'
Ngal	forward	5'-tggccctgagtgtcatgtg-3'
	reverse	5'-ctcttgtagctcatagatggtgc-3'
18s	forward	5'-gatccattggagggcaagtct-3'
	reverse	5'-aactgcagcaactttaatatacgctatt-3'
Gapdh	forward	5'- aactttggcattgtggaagg-3'
	reverse	5'-acacattgggggtaggaaca-3'

S4. Kidney TAG analysis. Total lipid extraction was performed on the kidneys using to the method of Folch, Lees and Sloane Stanley [6]. Briefly, tissue was homogenized in chloroform:methanol before the addition of sodium phosphate. The organic layer was dried down and lipids were then resuspended in chloroform, spotted onto a silica gel G plate alongside standards and resolved by thin layer chromatography. Bands corresponding to TAG were identified with the standards and scraped for quantification by gas chromatography as we have previously described [7].

S5. Tissue sectioning, Oil Red O staining, and H&E staining. To visualize renal accumulation of neutral lipids, frozen sections derived from kidneys embedded in OCT were stained with Oil Red O Stain (Cat #ab150678; Abcam, Toronto, Ontario, Canada), according to the manufacturer's instructions. Briefly, kidney slices were fixed in 10% formalin and dipped in propylene glycol, before incubation in Oil red O. Slices were then dipped in 85%

propylene glycol, rinsed and incubated in hematoxylin. The stained sections were imaged with an Axio Observer Z1 microscope (Carl Zeiss) and scored in a blinded manner.

References for Supplementary Methods

1. Ahmadian, M., et al., *Desnutrin/ATGL is regulated by AMPK and is required for a brown adipose phenotype.* Cell Metab, 2011. **13**(6): p. 739-48.
2. Tang, T., et al., *Desnutrin/ATGL activates PPARdelta to promote mitochondrial function for insulin secretion in islet beta cells.* Cell Metab, 2013. **18**(6): p. 883-95.
3. Shao, X., S. Somlo, and P. Igarashi, *Epithelial-specific Cre/lox recombination in the developing kidney and genitourinary tract.* J Am Soc Nephrol, 2002. **13**(7): p. 1837-46.
4. Breiderhoff, T., et al., *Deletion of claudin-10 (Cldn10) in the thick ascending limb impairs paracellular sodium permeability and leads to hypermagnesemia and nephrocalcinosis.* Proc Natl Acad Sci U S A, 2012. **109**(35): p. 14241-6.
5. Chen, J., et al., *Deficiency of FLCN in mouse kidney led to development of polycystic kidneys and renal neoplasia.* PLoS One, 2008. **3**(10): p. e3581.
6. Folch, J., M. Lees, and G.H. Sloane Stanley, *A simple method for the isolation and purification of total lipides from animal tissues.* J Biol Chem, 1957. **226**(1): p. 497-509.
7. Bradley, R.M., et al., *Acylglycerophosphate acyltransferase 4 (AGPAT4) is a mitochondrial lysophosphatidic acid acyltransferase that regulates brain phosphatidylcholine, phosphatidylethanolamine, and phosphatidylinositol levels.* Biochim Biophys Acta, 2015. **1851**(12): p. 1566-76.
